# Genetic deletion of microRNA-22 blunts the inflammatory transcriptional response to status epilepticus and exacerbates epilepsy in mice

**DOI:** 10.1186/s13041-020-00653-x

**Published:** 2020-08-21

**Authors:** Luiz F. Almeida Silva, Cristina R. Reschke, Ngoc T. Nguyen, Elena Langa, Amaya Sanz-Rodriguez, Rogerio R. Gerbatin, Fernanda R. Temp, Mayara L. de Freitas, Ronan M. Conroy, Gary P. Brennan, Tobias Engel, David C. Henshall

**Affiliations:** 1 Department of Physiology & Medical Physics, Royal College of Surgeons in Ireland, 123 St. Stephen’s Green, Dublin, D02 YN77 Ireland; 2 FutureNeuro SFI Research Centre, Royal College of Surgeons in Ireland, Dublin, Ireland; 3 Department of Physiology and Pharmacology, Federal University of Santa Maria, Santa Maria, RS Brazil; 4 Department of Epidemiology and Public Health Medicine, Royal College of Surgeons in Ireland, Dublin, D02 YN77 Ireland; 5 University College Dublin, Belfield, Dublin, Ireland

**Keywords:** Antagomirs, kainic acid, Hippocampus, microRNA, Oligonucleotide, Temporal lobe epilepsy

## Abstract

MicroRNAs perform important roles in the post-transcriptional regulation of gene expression. Sequencing as well as functional studies using antisense oligonucleotides indicate important roles for microRNAs during the development of epilepsy through targeting transcripts involved in neuronal structure, gliosis and inflammation. MicroRNA-22 (miR-22) has been reported to protect against the development of epileptogenic brain networks through suppression of neuroinflammatory signalling. Here, we used mice with a genetic deletion of miR-22 to extend these insights. Mice lacking miR-22 displayed normal behaviour and brain structure and developed similar status epilepticus after intraamygdala kainic acid compared to wildtype animals. Continuous EEG monitoring after status epilepticus revealed, however, an accelerated and exacerbated epilepsy phenotype whereby spontaneous seizures began sooner, occurred more frequently and were of longer duration in miR-22-deficient mice. RNA sequencing analysis of the hippocampus during the period of epileptogenesis revealed a specific suppression of inflammatory signalling in the hippocampus of miR-22-deficient mice. Taken together, these findings indicate a role for miR-22 in establishing early inflammatory responses to status epilepticus. Inflammatory signalling may serve anti-epileptogenic functions and cautions the timing of anti-inflammatory interventions for the treatment of status epilepticus.

## Introduction

Prolonged or repeated seizures are damaging to the brain and can establish lasting states of hyperexcitability that produce recurrent spontaneous seizures (epilepsy) [1, 2]. Gene expression programmes drive many of the changes underlying network reorganisation in epileptogenesis, including neurodegeneration, astrogliosis, microgliosis, aberrant neurogenesis and restructured local and distant neuronal contacts, among other changes [3–5]. A number of post-transcriptional mechanisms fine-tune the gene expression landscape. Small noncoding RNAs called microRNAs (miRNA) negatively regulate gene expression by sequence-specific targeting of protein-coding transcripts [6]. Binding of a miRNA to a complementary sequence in a target mRNA is mediated by Argonaute proteins leading to degradation of the mRNA or translational inhibition, thereby lowering protein levels in cells [7]. Individual miRNAs are capable of regulating gene networks by interacting with multiple targets or by suppressing transcriptional controllers [6, 8]. miRNAs are essential for brain development and function, with multiple miRNAs enriched in specific cell types where they regulate differentiation, structure and neurophysiological properties [9, 10].

Expression of various miRNAs is dysregulated in experimental models of status epilepticus and in resected brain tissue from patients with drug-resistant temporal lobe epilepsy [11–13]. This is predicted to exert an important influence on the gene expression landscape. Indeed, functional studies in animals, mainly using antisense oligonucleotides (ASOs) termed antagomirs, demonstrated that targeting miRNAs can alter evoked and spontaneous seizures and neuropathological outcomes including neuronal loss and gliosis [11]. miR-22-3p (hereafter miR-22), a conserved miRNA that is expressed throughout the body, including the brain [14, 15], was previously identified among upregulated miRNAs within the mouse hippocampus contralateral to the epileptogenic zone in the intraamygdala kainic acid model of status epilepticus [16]. Inhibiting miR-22 using intracerebroventricular injection of an ASO increased the frequency of spontaneous seizures, indicating that miR-22 has a protective role in the model [16]. Inhibition of miR-22 was also associated with increased astrogliosis [16]. The P2X7 receptor was identified as a miR-22 target and co-injection of a P2X7 receptor antagonist mitigated the epilepsy phenotype in mice given the ASO inhibitor of miR-22 [16]. Over-expression of miR-22 has been reported to protect in models of brain injury and additional targets of miR-22 have been identified [17, 18].

ASOs produce only transient and incomplete knockdown of RNAs and have the potential to modulate targets with closely-related sequences [19]. Accordingly, the role of miR-22 in epileptogenesis remains incompletely resolved. Mice with a genetic ablation of miR-22 (miR-22^−/−^) were recently developed [20]. The mice are viable and develop normally but display increased sensitivity to haemodynamic stress, consistent with roles for miR-22 in the heart [20]. Accordingly, we sought to evaluate epilepsy development after status epilepticus in mice lacking miR-22. We characterised the morphology of the hippocampus, basic behaviour, tracked the development of epilepsy using continuous electroencephalogram (EEG) recordings and explored how miR-22 ablation affected gene expression. Our findings confirm that miR-22 serves an important role in protecting against epileptogenic injury from status epilepticus and reveal an unexpected role in promoting an inflammatory transcriptional landscape.

## Results

### Characterisation of mice lacking miR-22

**Fig. 1:**
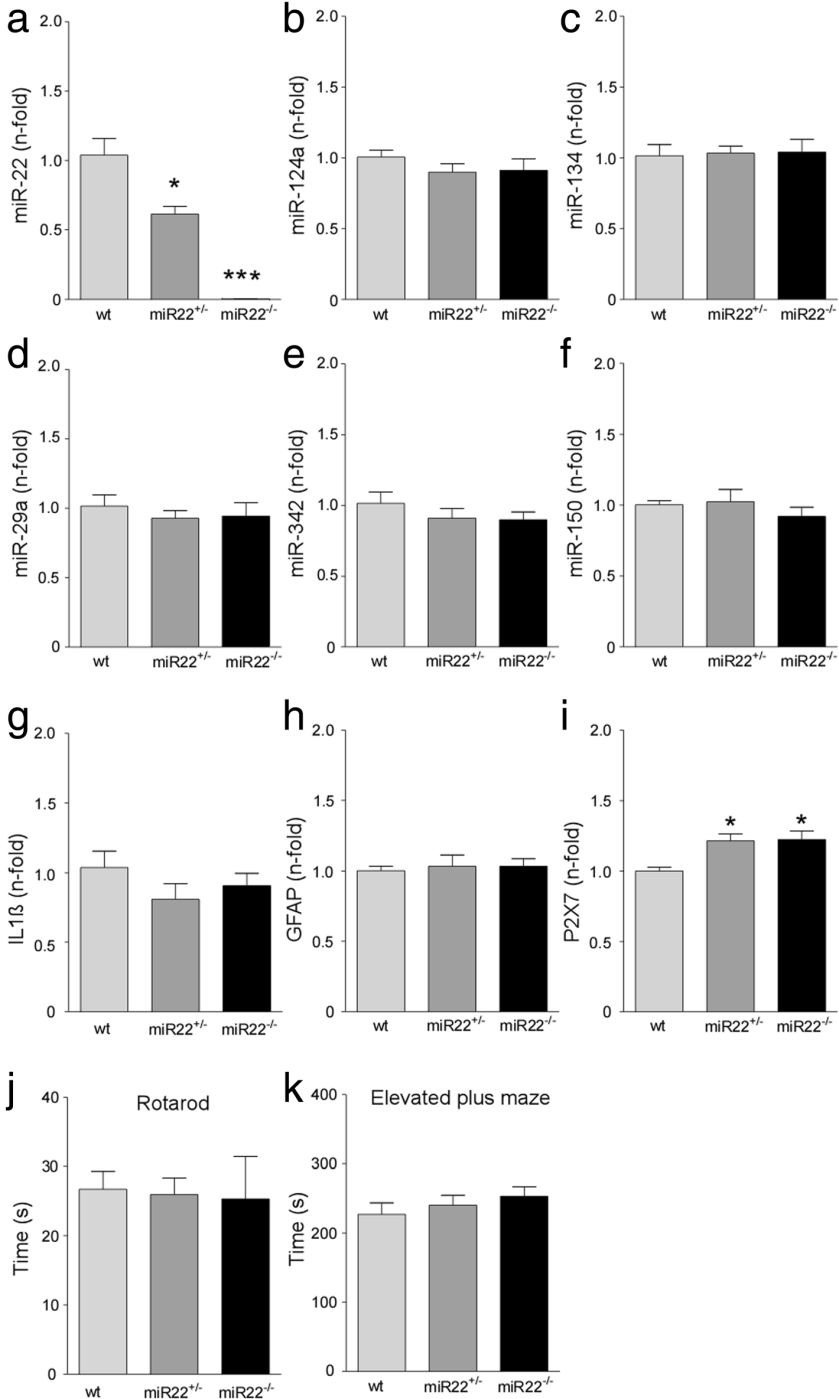
*miRNA expression and behaviour in miR-22-deficient mice.* (**a-e**) Graphs show relative expression of miR-22 and a selection of other brain cell type-enriched miRNAs in miR-22 mutant mice. **a** Graph confirms gene dose-related reductions of miR-22 in the hippocampus of miR-22^+/−^ and miR-22^−/−^ compared to wildtype (wt) mice. **b**-**f**. Expression of neuron-enriched miRNAs (miR-124a and − 134), microglial (miR-150, miR-342) and astrocyte (miR-29a) were unaltered in miR-22 mutant mice. **g**-**i** Graphs show expression of a selection of inflammation-related transcripts in the mutant mice. Note, slightly elevated expression of P2X7 which is a validated target of miR-22. **j**, **k** Graphs show miR-22-deficient mice perform normally in tests of coordination (rotarod) and anxiety (elevated plus maze). *P < 0.05 and ***P < 0.01 (*n* = 6/group; 3 males and 3 females)

We first analysed the expression of miR-22 and other miRNAs in the hippocampus of wildtype, heterozygous (miR-22^+/−^) and knockout (miR-22^−/−^) mice at 3 months of age. This confirmed a dose-dependent reduction in miR-22 levels consistent with heterozygous and full (homozygous) deletion of miR-22 (Fig. 1a). Since miR-22 is expressed in both neurons and glia [16], we explored whether loss of miR-22 results in compensatory changes to other miRNAs enriched in these cell types. Analysis of hippocampal levels of a selection of miRNAs including neuronal (miR-124-3p, miR-134-5p), astrocyte (miR-146a-5p, miR-29a) and microglial (miR-342, miR-150) miRNAs associated with roles in epilepsy [21–23] revealed normal levels of all tested miRNAs in the hippocampus of mice lacking miR-22 (Fig. 1b-f and Supplementary Data S1A). As we previously demonstrated that miR-22 targets the P2X7 receptor in the mouse hippocampus [16], we measured markers of inflammatory responses. Transcript levels of interleukin 1β (IL1β) and glial fibrillary acidic protein (GFAP) were not different in the hippocampus of naïve miR-22-deficient mice (Fig. 1g, h) whereas levels of P2X7 expression were slightly raised in miR-22-deficient mice (Fig. 1i), consistent with previous findings that miR-22 targets this transcript [16]. Analysis of the hippocampus from 9 months old miR-22^+/−^ and miR-22^−/−^ mice revealed no differences in levels of the tested miRNAs compared to wildtype (Supplementary Data S1B-G). Thus, loss of miR-22 does not result in compensatory changes to these cell-enriched miRNAs.

We also investigated the behaviour of miR-22-deficient mice. Mice lacking miR-22 did not display any locomotor impairments in the Rotarod test (Fig. 1j). Furthermore, miR-22^−/−^ mice performed similarly to wildtype mice in the elevated plus maze, an assay for anxiety-like behaviour (Fig. 1k). Attempts to assess hippocampal function using the Y maze forced alternation test were unsuccessful. This was due to prolonged periods of immobility and lack of exploration observed for all groups (Supplementary Data S1H-J), which has previously been reported for this background strain [24]. Sub-group analysis of male and female mice revealed there were no significant sex differences in any of these tests.

**Fig. 2:**
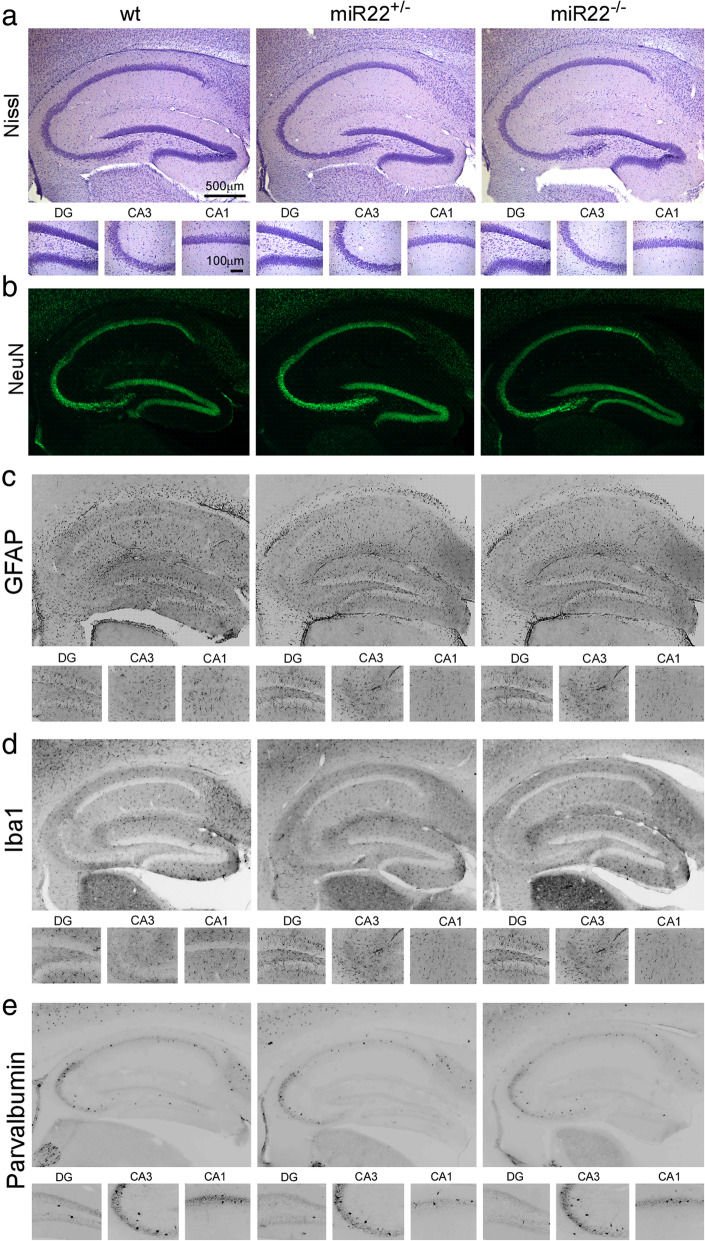
*Hippocampal histology in naïve mice lacking miR-22.* Representative photomicrographs showing morphology of the hippocampus of wildtype (wt) and miR-22-deficient mice (*miR-22*^*+/−*^, *miR-22*^*−/−*^). Sections were stained with markers of **a** nissl, **b** NeuN (neurons), **c** GFAP (astrocytes), **d** Iba1 (microglia) and **e** parvalbumin (interneurons). No differences were found for any parameter (*n* = 6/group)

Analysis of hippocampal tissue sections stained for Nissl (Fig. 2a) or the neuronal marker NeuN (Fig. 2b) revealed normal gross brain structure in 3 months old mice lacking miR-22. Staining and counts of GFAP positive cells, a marker of astrocytes (Fig. 2c), and Iba1, a marker of microglia (Fig. 2d), were normal in miR-22^−/−^ mice (and see Supplementary Data S2A, B with higher power images in S6). The morphology of these cells was not quantified. Staining and counts of parvalbumin inhibitory interneurons did not differ between genotypes (Fig. 2e and Supplementary Data S2C). The macroscopic structure of the ventral hippocampus also appeared normal in the mutant mice (Supplementary Data S2D). Inspection of brains from older, 9 months old heterozygous and homozygous miR-22-deficient mice also revealed no differences in staining of the same neuronal and glial histological markers (Supplementary Data S3).

### Status epilepticus in mice lacking miR-22

Wildtype, miR-22^+/−^ and miR-22^−/−^ mice were next equipped for acute EEG recordings to assess seizure severity during status epilepticus triggered by intraamygdala kainic acid. This is a well-characterised model of prolonged seizures that recruits limbic circuits and causes damage to the ipsilateral hippocampus. Mice develop spontaneous recurrent seizures after a short latency period of 3–5 days [25].

**Fig. 3:**
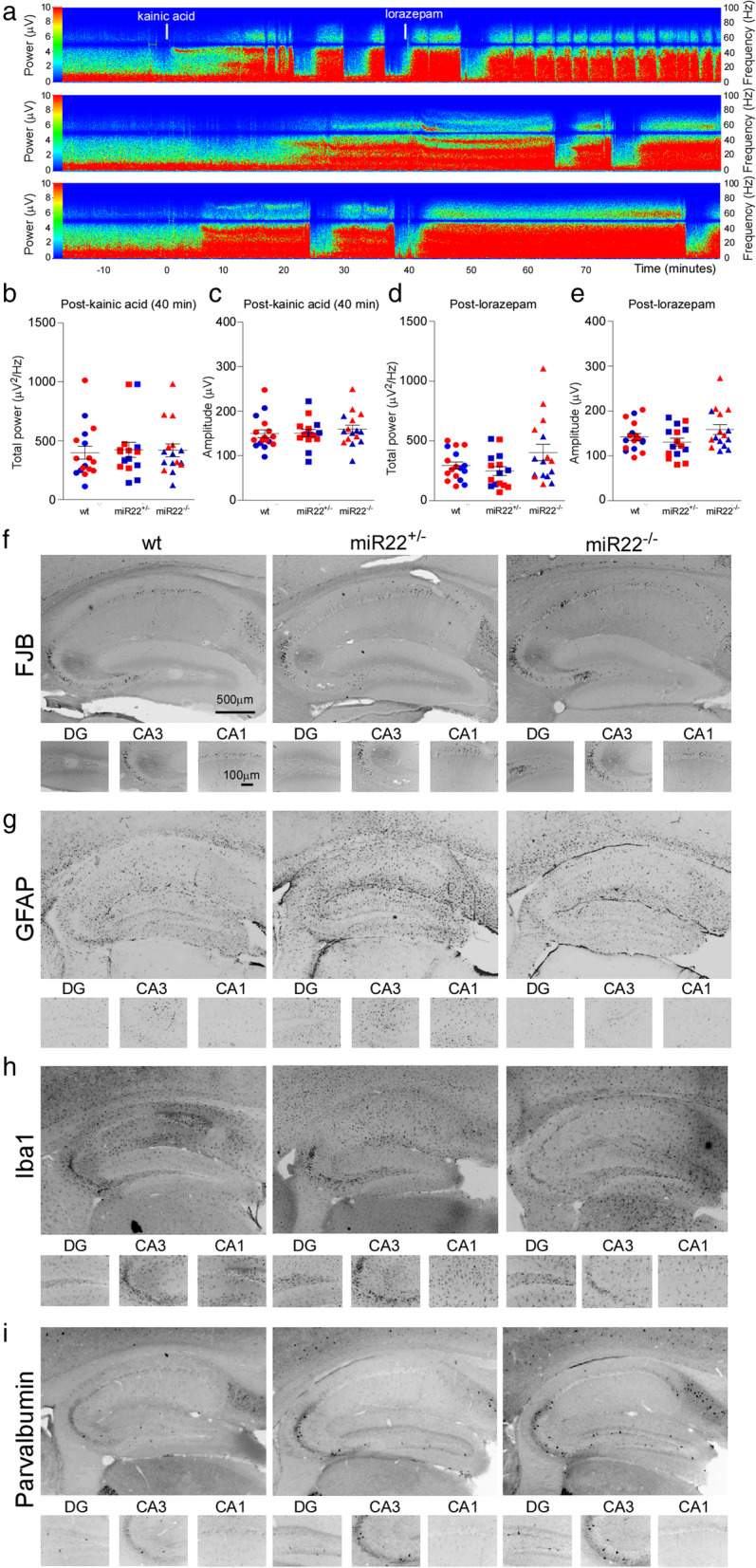
*Status epilepticus and acute histological outcomes in miR-22-deficient mice*. **a** Representative recordings of electrographic seizures (pseudocolour transforms of original EEG) during status epilepticus in mir-22^+/−^ (middle trace) and miR-22^−/−^ (bottom trace) mice compared to a wildtype (top trace) control. **b**-**e** Graphs show EEG total power and amplitude during the 40 min period from kainic acid administration to lorazepam and the 60 min period thereafter. Blue dots indicate male mice, red dots indicate female mice (*n* = 7–10/group). **f**-**i**. Representative photomicrographs of the hippocampus of mice 72 h after status epilepticus. There were no differences in acute neuronal loss, astrogliosis, microgliosis or interneuron (parvalbumin) staining

Status epilepticus after intraamygdala microinjection of kainic acid was similar between wildtype, miR-22^+/−^ and miR-22^−/−^ mice (Fig. 3a). Analysis of electrographic seizure activity and EEG parameters over the period between kainic acid and administration of anticonvulsant (to curtail seizures and reduce morbidity and mortality) revealed no difference amongst the groups or between sexes for EEG amplitude and total power (Fig. 3b, c). EEG parameters (amplitude and total power) also remained similar between groups after anticonvulsant administration (Fig. 3d, e).

Analysis of brains obtained 72 h after status epilepticus revealed similar neuronal damage and glial responses between wildtype, miR-22^+/−^ and miR-22^−/−^ mice (Fig. 3f-i and Supplementary Data S4). This included loss of neurons within the ipsilateral CA3 subfield of the hippocampus (Fig. 3f and Supplementary Data S4A). Staining for gliosis using GFAP and Iba1 was also similar between groups (Fig. 3g, h and Supplementary Data S4B, C and higher power images in S6). Parvalbumin-positive interneuron staining was also not different between genotypes (Fig. 3i and Supplementary Data S4D). Neuron counts within the injected amygdala were also similar between genotypes (Supplementary Data S4E). Taken together, these results indicate that genetic deletion of one or both copies of miR-22 did not affect acute electrographic and histopathological outcomes of status epilepticus in mice.

### Accelerated and exacerbated epilepsy phenotype in mice lacking miR-22

Next, wildtype and miR-22^−/−^ mice were equipped with implantable EEG telemetry units to record the long-term consequences of miR-22 deletion. Heterozygous mice were not included in further studies. After recovery from the surgery and induction of status epilepticus, mice were monitored 24 h per day for 14 days after status epilepticus.

**Fig. 4:**
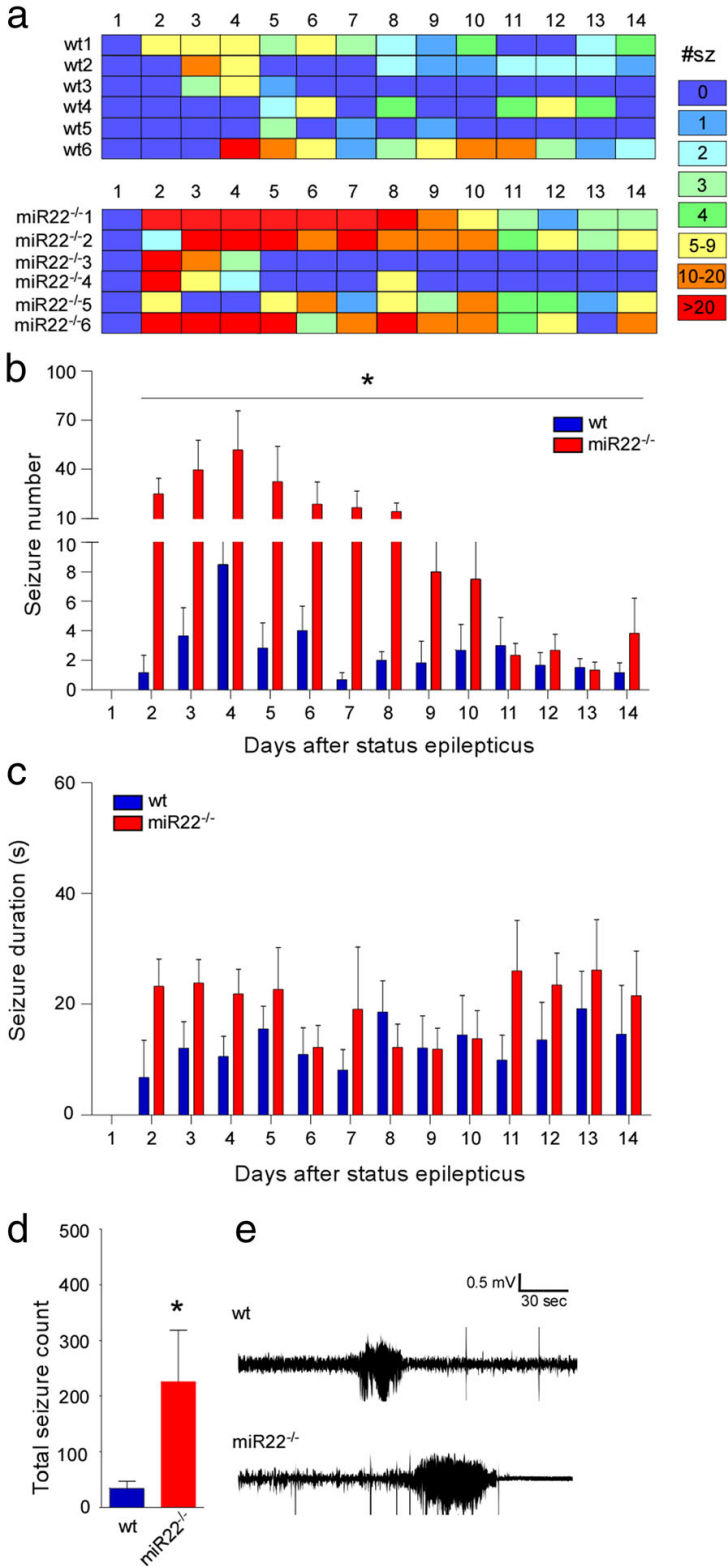
*Epilepsy phenotype in miR-22-deficient mice.* **a** Graphic shows individual mouse (1–6) daily seizure counts and summative graphs showing spontaneous seizures during 14 days recording in male wildtype (wt) and miR-22^−/−^ mice. **b** Graph showing numbers of spontaneous recurrent seizures during the 14 days recording in which miR-22^−/−^ mice display an elevated number of spontaneous seizures compared to wildtype mice. **c** Average time in seizures per day was increased in mice lacking miR-22 and average spontaneous seizure duration is longer in miR-22^−/−^ mice. **d** Total seizure counts for the two groups over the 14 days monitoring. *P < 0.05 (*n* = 6/group). **e** Representative traces of spontaneous seizures captured by telemetry EEG in a wildtype and miR-22^−/−^ mouse. Note the longer duration of seizure typical to miR-22-deficient mice

All mice lacking miR-22 developed their first spontaneous seizures on the second day after status epilepticus, compared to wildtype mice of which only a single animal presented their first spontaneous seizure on the second day (Fig. 4)a. All other wildtype mice developed their first spontaneous seizures from the third day onwards, consistent with the known course of epilepsy in the model [25] (Fig. 4a). A zero-inflated Poisson regression model was used to analyse the incidence of seizures. Statistical analysis showed that the incidence rate ratio of seizures was five times higher in the miR-22^−/−^ mice compared to wildtype animals. The average number of seizures per day in wildtype mice was 2.47 (range 1–31). In contrast, the average number of seizures per day in miR-22^−/−^ mice was 16 (range 1–138) (Fig. 4a, b). Statistical analysis also showed that miR-22^−/−^ mice presented on average significantly longer seizures (18.4 s) compared to wildtype mice (11.9 s) (Fig. 4c, e). Furthermore, as expected, the total number of seizures in miR-22^−/−^ over the 14 day period was higher when compared to wildtype mice (Fig. 4d). Notably, this higher rate largely occurred during the first 10 days of monitoring, with rates during the last few days similar between groups.

**Fig. 5:**
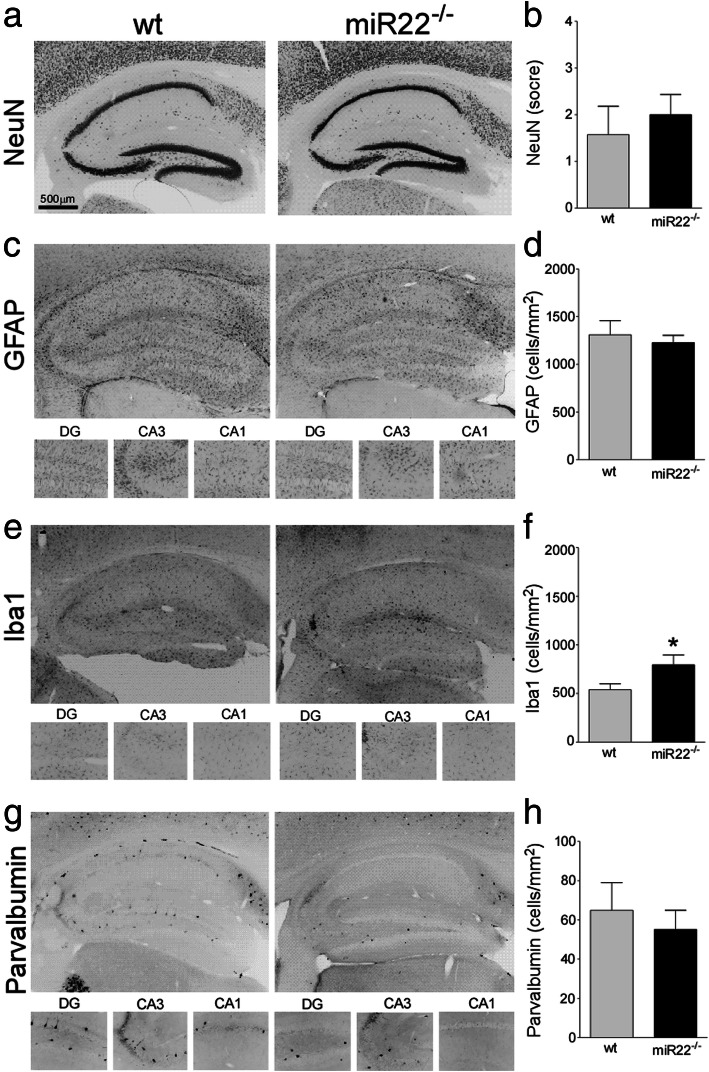
*Histological findings after epilepsy monitoring in mice lacking miR-22*. Representative photomicrographs and graphs of cell counts, showing histological outcomes in wildtype (wt) and miR-22-deficient mice at the end of 14 days epilepsy monitoring. **a**, **b** Neuronal loss, **c**, **d** astrogliosis, **e**, **f** microgliosis and **g**, **h** interneuron counts. Note, most cell markers were similar but microglial staining was increased in miR-22-deficient mice. *P < 0.05 (*n* = 6/group)

Analysis of brain tissue sections after epilepsy monitoring revealed similar overall histological changes. This included a macroscopic lesion comprising neuron loss and gliosis within the ipsilateral CA3 subfield (Fig. 5a, b). Counts of GFAP (Fig. 5c, d) and parvalbumin-positive cells (Fig. 5g, h) were similar between wildtype and miR-22^−/−^ (and Supplementary Data S5). In contrast, there was a small but significantly higher number of Iba1-stained microglia in miR-22^−/−^ mice at the end of monitoring (Fig. 5e, f and Supplementary Data S5 and higher power images in S6).

### RNA sequencing analysis of miR-22^−/−^ mice reveals down-regulation of inflammatory signalling pathways during epileptogenesis

Last, we sought insights into the molecular mechanism underlying the exacerbated epilepsy phenotype in miR-22^−/−^ mice. Previous studies identified the P2X7 receptor as a key target of miR-22, indicating a role in suppressing inflammation [16]. RNA sequencing was performed to identify dysregulated protein-coding mRNAs within the hippocampus 24 h after status epilepticus in wildtype and miR-22^−/−^ mice. This time-point captures the gene expression landscape after the epilepsy-inciting event (status epilepticus) but immediately before the emergence of the exacerbated epilepsy phenotype. Thus, differences can be more confidently attributed to the lack of miR-22 and are not obscured by possible effects of the differences in spontaneous seizures.

**Fig. 6:**
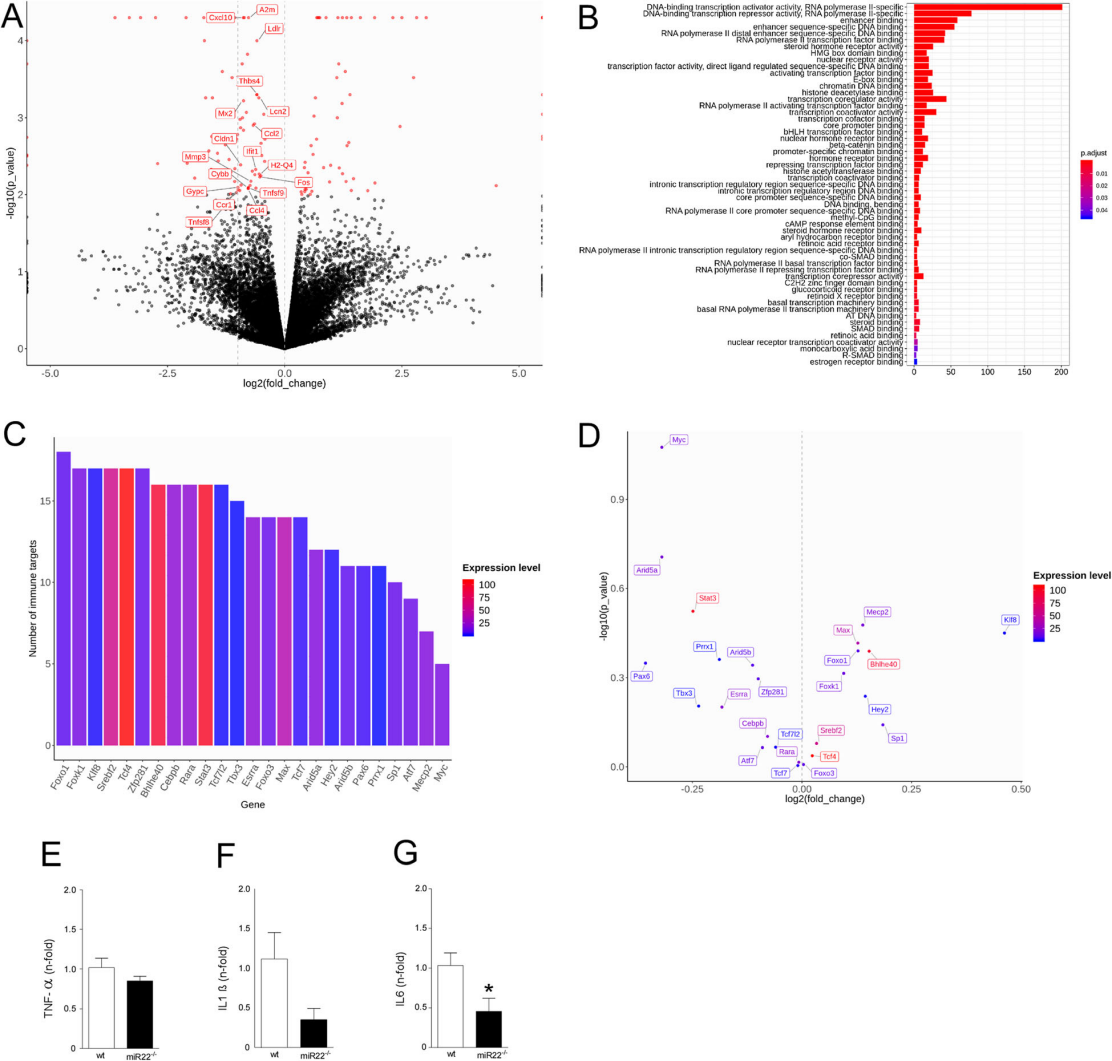
*RNA sequencing reveals downregulated inflammatory transcriptional landscape in miR-22-deficient mice*. **a** Volcano plot based on RNA sequencing, shows that miR-22 deficiency in mice resulted in the decreased expression of 18 immune-associated genes (labelled with names) in the hippocampus 24 h after status epilepticus. **b** Bar plot shows the numbers of genes in different classes of 437 transcription factors identified for the 18 immune-associated genes, including 93 transcription repressors and co-repressors (21.3%). **c** Bar plot showing the numbers of the immune-associated genes targeted by each of the 25 miR-22 mediated transcriptional repressors. Each of these repressors regulates several the immune-associated targets. **d** Volcano plot showing gene expression, magnitude of changes and statistical significance of differential expression analysis of the 25 transcriptional repressors. None of the repressors passed the statistical significance threshold (*P* value < 0.01) due to small magnitudes of changes in gene expression and/or relatively low expression abundance. (**e-g**) Quantitative PCR analysis of a selection of inflammation-related transcripts using the samples that were sequenced. *P < 0.05 compared to wt

We prioritised our bioinformatics search on mRNAs with shared functions and displaying small fold changes relevant to how miRNAs affect levels of their targets, to identify biologically relevant pathways dysregulated by loss of miR-22 [9] [8]. This revealed a set of 18 genes in inflammatory signalling-enriched pathways that exhibited small differences in gene expression levels between the wildtype and miR-22 ^−/−^ mice after status epilepticus. Remarkably, the transcript levels of all the genes were downregulated in the miR-22-deficient mice (Fig. 6a). A number of significantly enriched gene ontology (GO) terms of transcription factors regulating all these down-regulated immune-associated transcripts is shown in Fig. 6b, including 93 transcription repressors and corepressors. Among these (co)-repressors, 25 (27%) of them are putative targets of miR-22. There was an extensive overlap among the immune-associated targets of these transcriptional (co)-repressors (Fig. 6c), indicating these immune-associated genes are tightly controlled by miR-22 through the simultaneous manipulation of multiple transcriptional (co)-factors. However, expression of the individual repressors was not statistically different in miR-22 deficient mice, perhaps due to their low expression abundance and/or small magnitude of changes in expression levels (Fig. 6d). A selection of the inflammation-related pathways transcripts predicted to be down-regulated were measured using quantitative PCR and results were consistent with sequencing findings (Fig. 6e-g).

## Discussion

Here we show that genetic ablation of miR-22 results in a temporally-restricted, accelerated and exacerbated epilepsy phenotype in a mouse model of status epilepticus. Unexpectedly, the transcriptional landscape generated by loss of miR-22 featured extensive downregulation of inflammatory signalling pathways. These findings suggest miR-22 may activate as well as suppress neuroinflammation. Together, this extends the evidence for miRNA roles in the pathogenesis of epilepsy, imposes limits on the therapeutic potential of targeting miR-22, and cautions the delivery or timing of anti-inflammatory interventions for prevention of epileptogenesis after status epilepticus.

There is increasing evidence that miRNAs regulate the gene expression landscape during and after epileptogenic injuries [11, 13]. Here, we focused on miR-22 which is an abundant miRNA expressed in multiple tissues and cell types. Previous studies suggested miR-22 is protective in models of brain injury [17, 18], and ASO targeting demonstrated that inhibiting miR-22 exacerbated epilepsy, an effect due in part to de-repression of the P2X7 receptor [16]. Targeting using ASOs, however, produces only incomplete and temporary knockdown of miRNAs, leaving questions about the significance and potential therapeutic value of this miRNA for epilepsy. We addressed this limitation by studying the effect of genetic deletion of miR-22 on epilepsy phenotypes and molecular outcomes of status epilepticus.

The main finding in the present study was that genetic ablation of miR-22 results in exacerbated epilepsy in mice. Specifically, spontaneous seizures emerged earlier, occurred more frequently and were longer in animals lacking miR-22 in the intraamygdala kainic acid model. We can exclude this being due to a difference in the initial precipitating injury because, consistent with targeting miR-22 using ASOs [16], genetic deletion of miR-22 did not affect the status epilepticus. To the best of our knowledge, this is the first report showing deletion of a single miRNA in a model of status epilepticus results in an altered epilepsy phenotype. The finding supports a role for miR-22 in suppressing hyper-excitability in response to an epilepsy-precipitating injury [16, 26]. The accelerated and exacerbated phenotype in miR-22^−/−^ mice matches the overall findings from studies that used ASOs to reduce miR-22 levels in the same model [16]. However, the number of seizures recorded in miR-22-deficient mice in the present study is approximately double the number in the ASO study. This likely reflects the partial and transient reduction in miR-22 levels achieved using ASOs and is evidence of a gene dose-dependent effect of the loss of miR-22 accelerating and exacerbating the epilepsy phenotype. It is noteworthy that the exacerbated epileptic phenotype in miR-22^−/−^ mice was time-restricted and did not persist for the full two-week recordings. This indicates the most important influence of miR-22 in this model is during the epileptogenic phase or during the emergence of first spontaneous seizures. The seizure rates in miR-22^−/−^ mice appeared to normalise later in the second week of recordings. Future studies could explore if this is permanent and due to a compensatory change within the hippocampus of miR-22^−/−^ mice. For example, via neurophysiological adjustments, which could be assessed by molecular or electrophysiological studies around the time when seizure rates normalise. Since spontaneous seizures were more frequent and lasted longer in miR-22-deficient mice, miR-22 may have targets that influence ictogenesis and seizure termination, the mechanisms of which remain incompletely understood [27]. More broadly, the findings may be clinically relevant since miR-22 is expressed within the hippocampus of patients with drug-resistant epilepsy [26].

The observation that genetic deletion of miR-22 does not affect susceptibility to status epilepticus is consistent with our observation that miR-22^−/−^ mice lack any obvious brain abnormalities and the known viability of the mice from their original description [20]. Indeed, neuronal and glial numbers and distribution appeared normal in naïve miR-22^−/−^ mice and animals behaved normally. Thus, despite its relative abundance, miR-22 is probably dispensable for normal brain organisation. This contrasts with the overt brain phenotypes of mice lacking certain other brain-enriched miRNAs which include gross brain development abnormalities, neuronal migration defects and epilepsy [28, 29]. The result is consistent with ASO targeting of miR-22 which did not alter the duration or severity of status epilepticus in the same model [16]. Histopathological outcomes after status epilepticus including neuronal loss and gliosis were also equivalent between miR-22^−/−^ and wildtype mice and only a modest difference in microgliosis was observed in the hippocampus at the end of epilepsy monitoring. This is consistent with animals having experienced an equivalent status epilepticus and with the similar final rates of spontaneous seizures between groups at the end of recordings. These findings differ somewhat from those reported when miR-22 was blocked using an ASO [16]. In that study, increased spontaneous seizures occurred later and in association with increased astrogliosis. This discrepancy may arise from cellular responses to transient, uneven and incomplete reduction of miR-22 levels using ASOs compared to complete deletion in the knockout mice. As miR-22^−/−^ mice experienced more spontaneous seizures than ASO-treated mice, microglial rather than astroglial increases may be a cellular correlate of the cumulative or overall burden of spontaneous seizures in this model. This would be consistent with microglia responses and recent seizures in humans [30], the use of microglial-based brain imaging as epilepsy biomarkers [31, 32] and experimental evidence that microglia changes are epileptogenic [33]. Notably, the microglial changes may be unrelated to inflammatory responses since microglial perform other roles including synaptic pruning [34].

The present study used RNA sequencing to define the transcriptional landscape that may be driving the altered epilepsy phenotype in mice lacking miR-22. A number of known miR-22 targets have been linked to epilepsy, including the purinergic P2X7 receptor that drives neuroinflammation through releasing IL1β [16]. Unexpectedly, RNA sequencing analysis of the hippocampus revealed that the gene expression landscape of mice lacking miR-22 is enriched with downregulated transcripts linked to inflammation. This included multiple transcripts involved in interleukin, cytokine, TNF and NF-kB signalling, all contributing to the regulation of neuroinflammatory responses. This finding reveals a more complex and nuanced function of this miRNA, regulating the outcome of status epilepticus by both strongly suppressing individual pro-inflammatory targets such as the P2X7 receptor as well as smaller effects across multiple transcripts that promote inflammation. This is consistent with the dual and opposing roles that have emerged for other brain enriched miRNAs [22] and updates concepts of how miRNAs regulate brain excitability and epilepsy [11].

Why did we see extensive down-regulation of inflammatory transcripts in miR-22^−/−^ mice? Because miRNAs largely function as negative regulators of gene expression [6, 8], loss of a miRNA will typically de-repress and upregulate targets. The RNA sequencing findings can be explained if one or more miR-22 target is a transcriptional repressor which upon de-repression by deletion of miR-22 then shuts down genes under its control. Our bioinformatics analysis identified multiple potential transcriptional repressors suggesting that the downregulation of inflammatory pathways could be mediated by de-repression of a transcriptional silencer. Notably, transcriptional silencers of inflammation have previously been identified for miR-22 including *Hdac4* [35]. Although *Hdac4* was not found among the increased genes in miR-22^−/−^ mice, the present study identified several other plausible candidates. Future studies will be needed to identify which such miR-22 targets target the transcriptional control elements of the down-regulated gene networks and whether this is causally involved epileptogenesis.

The present findings challenge the perceived pathogenicity of neuroinflammation in epileptogenesis. Certainly, prolonged or excessive cytokine signalling and neuroinflammation is a causal mechanism in the development and maintenance of the epileptic state [5]. But if epilepsy develops earlier and is more severe in mice with a subdued inflammatory transcriptional landscape then the neuroinflammatory response to status epilepticus, at least during the early period, is not maladaptive or pro-epileptogenic. This supports other evidence that early coordinated inflammatory signalling is a necessary and protective response to epileptogenic brain insults [5, 36]. Inflammatory signalling serves various protective functions including clearance of cellular debris after injury and prompting glial responses that contribute to repair [37]. This may complicate therapeutic strategies. Treatment with anti-inflammatory drugs, if mistimed or aligned with specific early phases, could lead to exacerbation of epileptogenesis. The finding that loss of miR-22 results in an accelerated epileptogenesis suggests delivery or over-expression of miR-22 might be a potential therapeutic strategy. This could be achieved using miRNA mimics, synthetic double-stranded molecules or other approaches [17, 18]. Notably, intracerebral injection of mimics for miR-22 and other inflammation-linked miRNAs has been reported to reduce seizures in the present model [16, 38].

There are caveats to consider in the present study. Foremost, we used a constitutive knockout mouse to study the effects of miR-22 deletion [20]. This is limiting because miR-22 is expressed in multiple cell types in the brain and the amounts of miR-22 expressed by cells differ between neurons and glia [16]. It will be important to understand whether the pathways controlled by miR-22 during epileptogenesis are cell type-specific. That is, does miR-22 exert its inflammatory actions in all or specific cells? The use of inducible knockout lines would allow the cell-specific and timed deletion of miR-22 from neurons and glia. While we did not observe differences in status epilepticus in the intraamygdala kainic acid model, miR-22-deficient mice may display altered vulnerability to other chemoconvulsants or epileptogenic injuries. Moreover, neuronal microstructure or subtle cellular dysorganisation may have been missed presently and recent work has shown that inhibiting miR-22 after status epilepticus produces changes to dendritic branching patterns in new hippocampal neurons [26]. Finally, a limit of the present study is that we did not perform a recovery experiment. It would be interesting in future studies to determine if the knockout phenotype we observed can be recovered or obviated by the reintroduction of miR-22. For example, testing if delivery of a mimic [16, 38] increases early inflammatory signalling and reduces the excess of spontaneous seizures in miR-22^−/−^ mice subject to status epilepticus.

In summary, the present study extends the evidence that miR-22 serves an important role in countering the epileptogenic process triggered by status epilepticus. Genetic deletion of miR-22 results in a temporally-restricted, accelerated and exacerbated epilepsy phenotype in the intraamygdala kainic acid model of status epilepticus and is associated with large-scale reduction in the transcriptional inflammatory response. The findings indicate early, protective effects of inflammatory responses during epileptogenesis and suggest miR-22 or the pathways under its control could be potential targets for the development of disease-modifying treatments.

## Materials and methods

### Breeding and identification of miR-22 knockout mice

Mice carrying a knockout (null) mutation of mir-22 were purchased from the Jackson laboratory (*Mir22*^*tm1.1Arod*^, Stock No: 018155) [39]. Mice were mated in trios (one male and two females). For breeding, either a single heterozygous male was mated with two homozygous mutant female mice or a single homozygous male mated with two heterozygous females. Wildtype mice were also mated in trios. Mice were housed in a climate-controlled biomedical facility on a 12-h light/dark cycle with food and water provided ad libitum. Primers for genotyping were (Forward (common), 5′ TGG GAC TTG GGT TCT ACA CC 3′; Reverse (wildtype), 5′ TCC TAA AAG GAA GGG GAG GA 3′; Reverse (miR-22 mutant), 5′ TGC TTT AGG TGG AGG GAA AG 3′.

### Status epilepticus and epilepsy monitoring

Status epilepticus was induced by unilateral intraamygdala microinjection of kainic acid, as described [16, 25]. Briefly, animals were anesthetized with isoflurane, placed in a stereotaxic frame and maintained normothermic by means of a feedback-controlled heat blanket. A craniotomy was performed and a guide cannula positioned over the surface of the dura. For acute EEG recordings, mice were equipped with three skull-mounted screw electrodes (Bilaney Consultants, U.K.) and connected to a Grass 40 channel lab-based digital EEG. For long-term epilepsy monitoring, mice were equipped with an EEG telemetry device (Model: F20-EET, Data Systems International) inserted subcutaneously and connected to skull screws. After baseline EEG recordings, a cannula was inserted to inject kainic acid (0.3 μg/0.2 μl in phosphate-buffered saline; PBS) or PBS into the basolateral amygdala nucleus. After 40 min, all mice received an intraperitoneal injection of lorazepam (8 mg/kg) to curtail seizures and reduce morbidity and mortality. EEG recordings during acute status epilepticus were analysed as before [23], including total power, amplitude and number of electrographic seizures. For long-term EEG monitoring, the number and duration of spontaneous seizures were manually scored, with epileptic seizures being defined as high-amplitude (>2X baseline) polyspike discharges of ≥10 s duration [16]. Mice were killed by pentobarbital overdose and perfused with PBS. Brains were then either microdissected and frozen or retained whole for sectioning.

### Rotarod, elevated plus and Y maze

The rotarod test was used to assess motor coordination and balance. After a habituation trial day, mice were placed on a horizontally oriented, rotating suspended cylinder and the length of time that mice stayed on the rotating rod was measured during a gradual acceleration from 0 to 40 rpm over 120 s. The average time each mouse spent on the accelerating rotating rod was calculated. The elevated plus maze apparatus comprised two open arms (25 cm length × 5 cm width) and two closed arms (25 cm length × 5 cm width × 16 cm height walls to enclose the arms) placed in the centre of the testing room, 50 cm above the floor. The test consisted of a single trial lasting 5 min. Mice were individually placed in the centre facing one of the open arms. The number of entries into the open and closed arms and the time spent was recorded. These parameters were used to calculate the index of anxiety-like behaviour. Spatial recognition memory test was conducted using a symmetrical Y-maze (35 cm × 5 cm × 60 cm) with the floor and walls made of white and clear Plexiglas respectively as previously described [40]. The mouse was placed into the end of the start arm, facing the wall and away from the centre. In the sample trial, mouse was allowed to explore during 5 min the two arms of the Y-maze, while entry into the third arm was blocked. After the sample trial, the mouse was returned to its home cage for a 60 min inter-trial interval. In the trial 2, the block in arm 3 (Novel arm) was removed and the mouse was again placed into the start arm, and then allowed to access all three arms of the maze. Time spent in Novel Arm was recorded only when 85% of a mouse’s body entered the arm and animals with less than three arm entries during the test trial were excluded from the analysis.

### Histopathology

Immunohistochemistry was performed as described [16], using post-fixed fresh frozen or paraformaldehyde-perfused sections. Sections were blocked in goat serum followed by incubation with primary antibodies against NeuN, glial fibrillary acidic protein (GFAP), ionized calcium binding adaptor molecule 1 (Iba1) or parvalbumin (all from Cell Signaling Technology). Negative controls were included that omitted the primary antibody. Slides were washed then incubated with secondary antibodies, rinsed again and mounted. Positive cells were quantified and were the average of two adjacent sections. One 20X picture was taken from each individual sub-field of the hippocampus (DG, CA3 and CA1). The numbers of positive cells as well the area were calculated and results were expressed as number of positive cells per mm^2^. Neuronal damage was semi-quantitatively assessed based on a graded five-point system under 40X magnification: Two independent researchers were blinded to experimental treatment and graded the neuronal damage in the CA3 sub-region as level 0, no damage; level 1, 0–25%; level 2, 25–50%; level 3, 50–75%; level 5, 75% cell loss, the average scoring of the 2 experimenters was calculated and analysed.

### Analysis of miRNA expression

Expression of individual miRNAs was performed using Taqman individual miRNA assays as described [16]. Levels of miR-22 and a selection of miRNAs enriched in neurons (miR-124a, miR-134), microglia (miR-342, miR-150) and astrocytes (miR-146a, miR-29a) was based on in situ hybridisation and in vitro cell culture studies [21–23, 41]. Five microliters of RNA (500 ng/μl) was mixed with RT buffer, RNAase inhibitor, multiscribe reverse transcriptase enzyme, dNTPs and the microRNA specific RT primer (Applied Biosystems) and placed in a thermocycler. Tested miRNAs were: miR-124a, miR-134, miR-150, miR-29a, miR-146a, miR-342. The generated cDNA was diluted, mixed with TaqMan Fast Universal PCR Master Mix and miRNA-specific PCR primers and ran in triplicate and analysed using the QuantStudio™ 12 K Flex PCR system as described [16].

### Analysis of mRNA expression

Expression of individual protein-coding gene transcripts was performed according to previous techniques [16]. Briefly, one microgram total RNA was used to generate cDNA by reverse transcription using Superscript II Reverse Transcriptase enzyme (Invitrogen). Quantitative real-time PCR was performed using a LightCycler 1.5 (Roche Diagnostics) in combination with QuantiTech SYBR Green PCR kit (Qiagen Ltd) as per manufacturer’s protocol and 1.25 μM of primer pair used Data were analyzed by LightCycler 1.5 software; data normalized to expression of β-Actin and represented at RQ values. Specific primers for each gene assayed were purchased from Sigma, and sequences used were as follows: β-Actin (Forward (F): gggtgtgatggtgggaatgg, Reverse: ggttggccttagggttcagg); Gfap (F: tatgaggaggaagttcgag, R: tgtctcttgcatgttactgg); IL1β (F: tgaagttgacggaccccaaa, R: agcttctccacagccacaat); P2x7 (F: ttggcaagatgtttctcgtg, R: actggcaggtgtgttccata);; Tnfα (F: ctcttcaagggacaaggctg, R: cggactccgcaaagtctaag); and Il6 (F: ctcagagtgtgggcgaacaa, R: actaactggaaggcttgccc).

### RNA sequencing preparation and analysis

Wildtype and miR-22^−/−^ mice (*n* = 4 each) were subjected to status epilepticus as described above. Twenty-four hours later, mice were euthanised and the ipsilateral hippocampus was obtained. Total RNA was extracted and library preparation was performed using an Illumina TruSeq Stranded mRNA Sample Prep Kit (poly-A enrichment). The generated RNA-Seq data were deposited in the Sequence Read Archive under GEO accession number GSE147466. Follow-up analysis including QC, alignment (reference genome GRCm38, Annotation_Ensembl70), mapping and raw analysis was performed by a service provider (Exiqon A/S, Vedbaek, Denmark). On average 41.3 million reads were obtained for each sample and the average genome mapping rate was 94.4%. Numbers of identified genes ranged from 21,260 to 22,315.

To reflect the mostly subtle effects of miRNAs on gene expression, only genes with small fold changes (less than two times, *P* < 0.01) were included in the pathway enrichment analysis. A total of 67 genes were searched against Reactome [42] and KEGG databases [43] using ReactomePA [44] and EnrichR [45]. Genes involved in immune-associated pathways of the top five most significantly (*P* < 0.05) enriched pathways from each analysis were selected and combined to analyse further. Potential transcription factors for these genes were retrieved from Tf2DNA database [46].

To identify targets of mmu-miR-22, experimentally validated targets were retrieved from miRTarBase Release 7.0 [47], TarBase v.8 [48] and miRecords [49] while predicted targets were retrieved from TargetScan Release 7.2 [50] and miRDB Version 6.0 [51] and processed as described previously [52], with some modifications. Briefly, prediction scores of TargetScan targets were rescaled between 0 and 1 while those of miRDB targets were rescaled between 0.5 and 1 (since original miRDB database excluded all targets with scores < 50). Then, any targets with rescaled prediction scores < 0.5 were removed from further analysis. All data processing, analyses and visualisation were performed using R and bash scripts in Unix working environment.

#### Data analysis

Data are presented as mean ± SEM. Comparison of data was performed using ANOVA with appropriate post hoc test. EEG variables (e.g., total power, frequency) were analysed using the parametric Student’s t-test, considering *P* < 0.05 as significant. For the long-term experiments, Zero-inflated Poisson regression was used to compare daily seizure counts between treatment groups. Robust variance estimation was used to correct for the effects of measurements within animals. Significance was accepted when *P* < 0.05.

## Supplementary information

**Additional file 1.**

## Data Availability

The dataset(s) supporting the conclusions of this article are included within the article and additional supplementary files and in the GEO submission.

## Abbreviations

ANOVA: Analysis of variance
ASO: Antisense oligonucleotide
EEG: Electroencephalogram
GFAP: Glial fibrillary acidic protein
GO: Gene ontology
PCR: Polymerase chain reaction
miRNA: MicroRNA
RNA: Ribonucleic acid
SEM: Standard error of the mean
wt: Wildtype
