# Supplementary material for: Genetic deletion of microRNA-22 blunts the inflammatory transcriptional response to status epilepticus and exacerbates epilepsy in mice
Source: Mol Brain. 2020 Aug 21;13:114. doi: 10.1186/s13041-020-00653-x (PMC7441563; doi:10.1186/s13041-020-00653-x)
Supplement: Supplementary file 1 — Additional file 1. [file 13041_2020_653_MOESM1_ESM.pdf]

Supplementary Data

Genetic deletion of microRNA-22 blunts the inflammatory transcriptional response to status epilepticus and exacerbates epilepsy in mice

Authors: Luiz F. Almeida Silva, Cristina R. Reschke, Ngoc T. Nguyen, Elena Langa, Amaya Sanz Rodriguez, Rogerio Gerbatin, Mayara L. de Freitas, Fernanda R. Temp, Ronan M. Conroy, Gary P. Brennan, Tobias Engel, and David C. Henshall

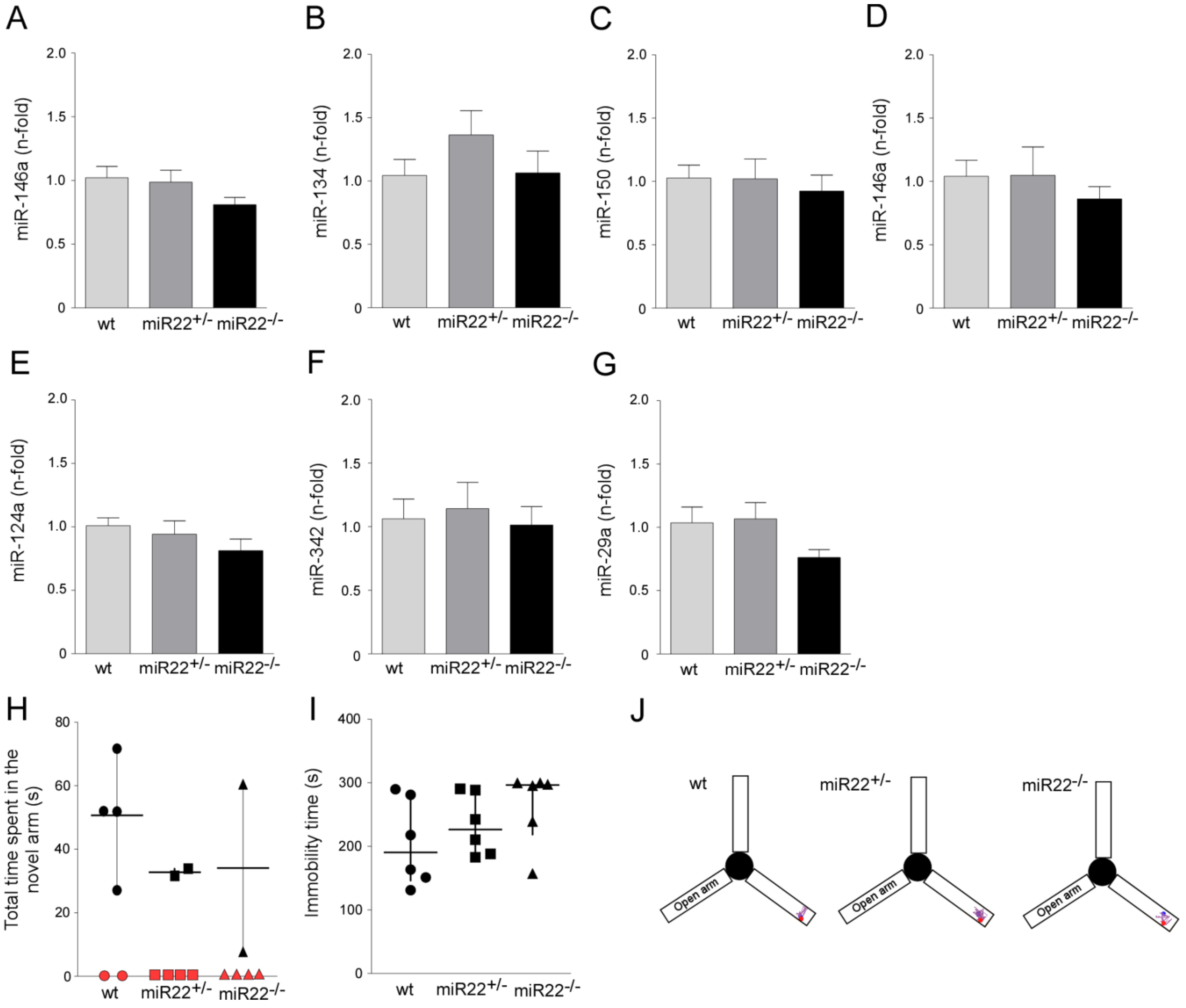
A
miR-146a (n-fold)
2.0
1.5
1.0
0.5
0
wt
miR22+/-
miR22-/-
B
miR-134 (n-fold)
C
miR-150 (n-fold)
D
miR-146a (n-fold)
E
miR-124a (n-fold)
F
miR-342 (n-fold)
G
miR-29a (n-fold)
H
Total time spent in the novel arm (s)
80
60
40
20
0
I
Immobility time (s)
400
300
200
100
J
Open arm

Supplementary Data Figure S1. *miRNA expression in the hippocampus of naïve miR-22-deficient mice and behaviour of miR-22-deficient mice.* (A) Graph shows relative expression of miR-146a in three months old miR-22 mutant mice (*n* = 6/group; 3 males and 3 females). (B-G). Graphs show relative expression of a selection of brain cell type-enriched miRNAs in miR-22 mutant mice at nine months of age. Expression of neuron-enriched miRNAs (miR-124a and -134), (microglial (miR-150, miR-342) and astrocyte (miR-146a, miR-29a) were unaltered in older miR-22 mutant mice. (H-J) Results of testing mice in a hippocampal-dependent tasking (forced alternating Y maze. Results show no difference between the mice in this task. However, the test was compromised by the limited movement and exploration of the mice in the assay. (H) Graph shows time spent in novel arm and animals which did not meet the minimum exploratory criteria are marked in red and were excluded from the analysis. (I) Graph shows limited exploratory behaviour with animals presenting increased immobility time across all groups. (J) Representative image of the exploratory behaviour, blue dot indicates beginning of the test and red dot indicates the end. (*n* = 6/group; 3 males and 3 females).

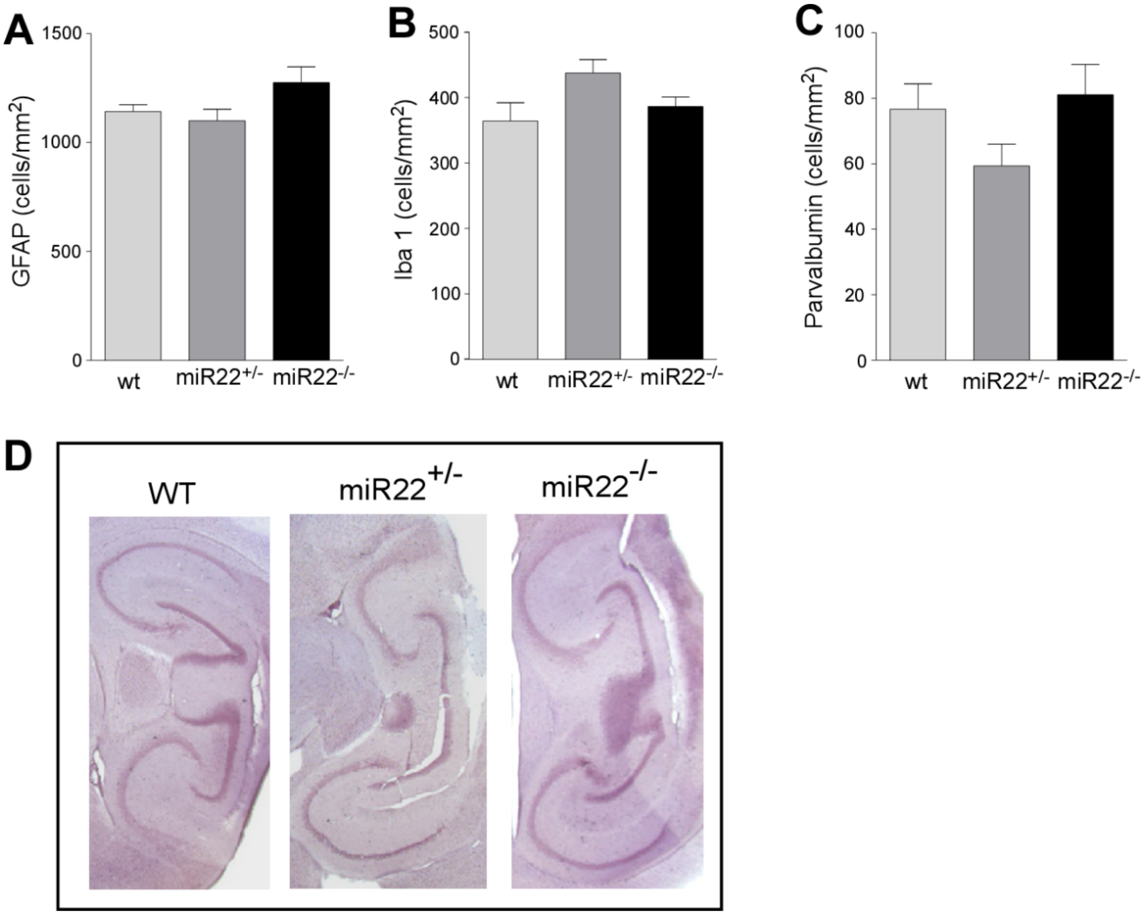

Supplementary Data Figure S2. *Histological findings in naïve mice lacking miR-22.* (A-C) Graphs showing counts of different cell types in the hippocampus of three months old wildtype (wt) and miR-22-deficient mice (*n* = 6 per group). Sections were stained with markers of astrocytes (GFAP), microglia (Iba1) and interneurons (parvalbumin). No differences in counts were found for any cell type. (D) Nissl-stained images of the hippocampal structure showing both ends from sagittal-plane sectioned naïve brains of each genotype.

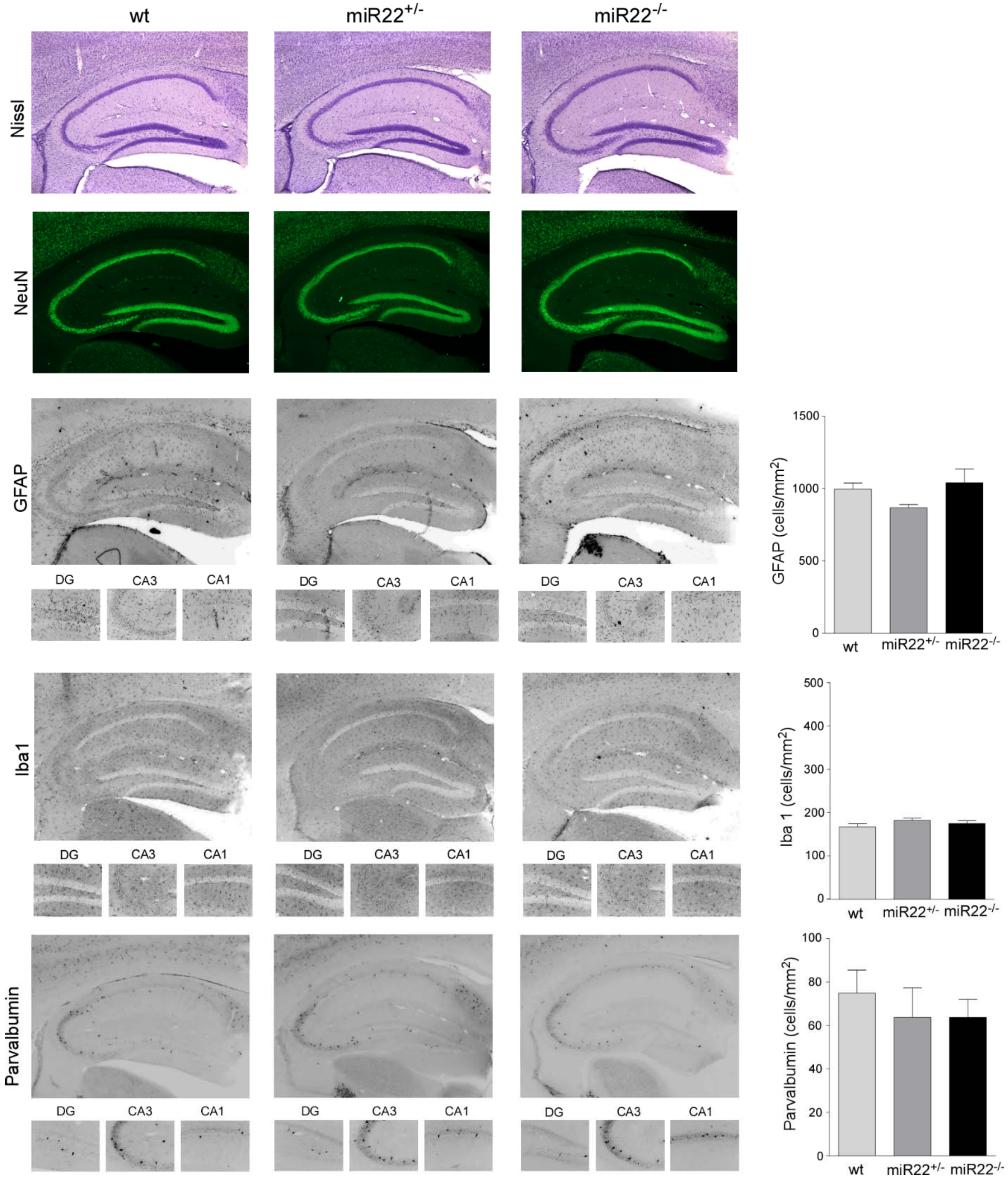
wt
miR22+/-
miR22-/-
Nissl
NeuN
GFAP
DG
CA3
CA1
GFAP (cells/mm2)
1500
1000
500
0
wt
miR22+/-
miR22-/-
Iba1
Iba 1 (cells/mm2)
500
400
300
200
100
0
Parvalbumin
Parvalbumin (cells/mm2)
100
80
60
40
20
0

Supplementary Data Figure S3 (see above). *Histological findings in nine months old naïve mice lacking miR-22.* Representative photomicrographs showing morphology of the hippocampus of nine months old wildtype (wt) and miR-22-deficient mice (*n* = 6 per group). Sections were stained with markers of nissl, NeuN (neurons), GFAP (astrocytes), Iba1 (microglia) and parvalbumin (interneurons). Graphs (Left) depict counts of cells (n = 6 per group; no statistical differences).

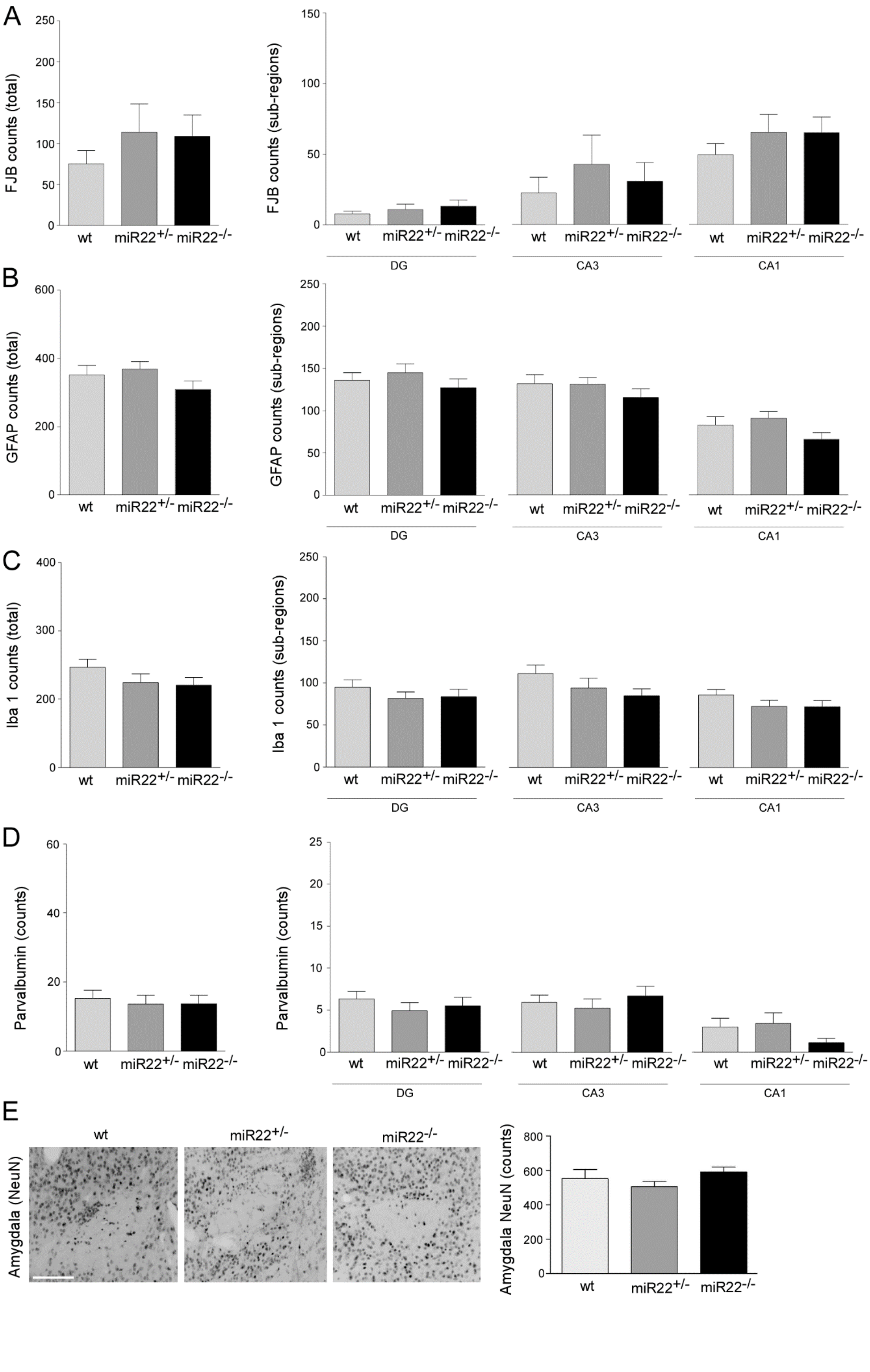
A
FJB counts (total)
FJB counts (sub-regions)
B
GFAP counts (total)
GFAP counts (sub-regions)
C
Iba 1 counts (total)
Iba 1 counts (sub-regions)
D
Parvalbumin (counts)
E
Amygdala (NeuN)
Amygdala NeuN (counts)
wt
miR22+/-
miR22-/-
DG
CA3
CA1

Supplementary Data Figure S4 (see above). *Acute histological outcomes in miR-22-deficient mice after status epilepticus*. (A) Graphs show counts of Fluorojade B (FJB) positive cells, a marker of neurodegeneration in the whole hippocampus (left) and subfields (right graphs), 72 h after status epilepticus in each genotype. (B - D) Counts of (B) GFAP, (C) Iba1 and (D) parvalbumin positive cells in sections 72 h after status epilepticus. *n* = 7 – 10 per group. (E) Assessment of the amygdala in tissue sections from each genotype after status epilepticus. Sections were stained for a neuronal marker (NeuN) and the total number of positive cells were counted within a 20X magnification field over the intraamygdala injection site (*n* = 4 - 6 per group). Bar, 100 μm. There were no differences in acute neuronal loss, astrogliosis, or microgliosis or in the size of the amygdala.

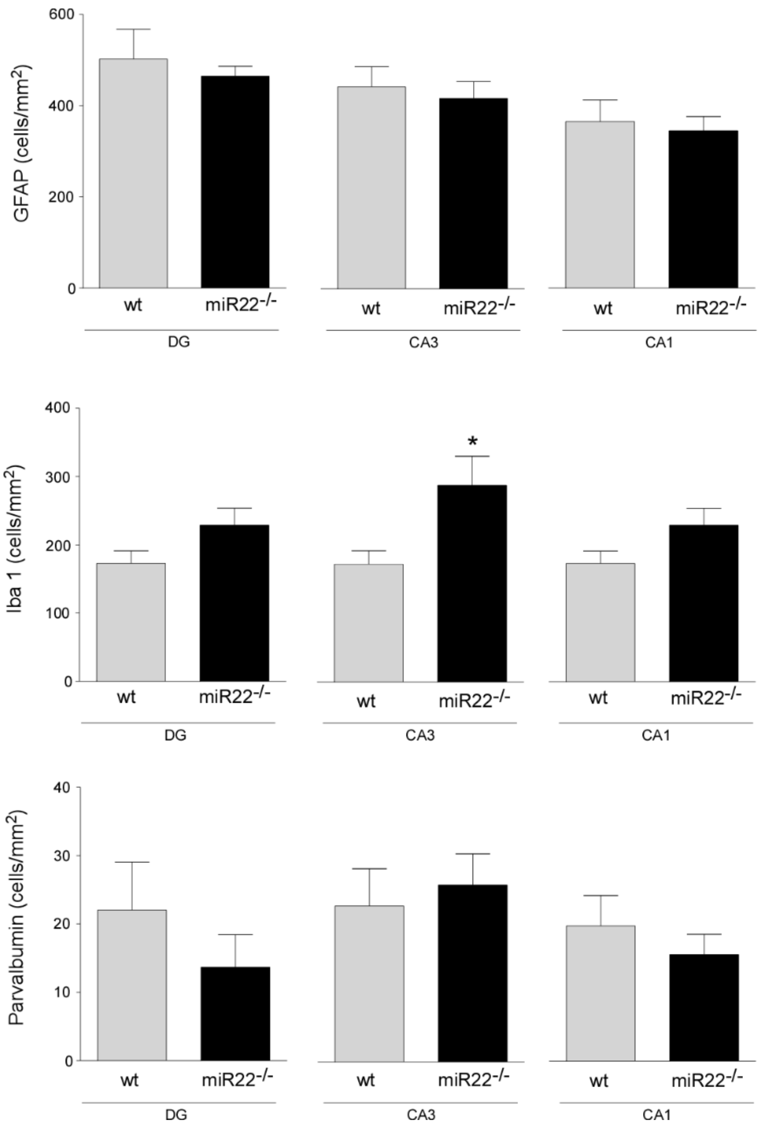

Supplementary Data Figure S5. *Histological findings after epilepsy monitoring in mice lacking miR-22*. Graphs show counts of hippocampal tissue sections stained for markers of (Top) astrogliosis, (Middle) microgliosis and (Bottom) interneurons. Counts were similar except for Iba1 staining which was increased in the CA3 subfield. *$P < 0.05$ ($n = 6$ per group).

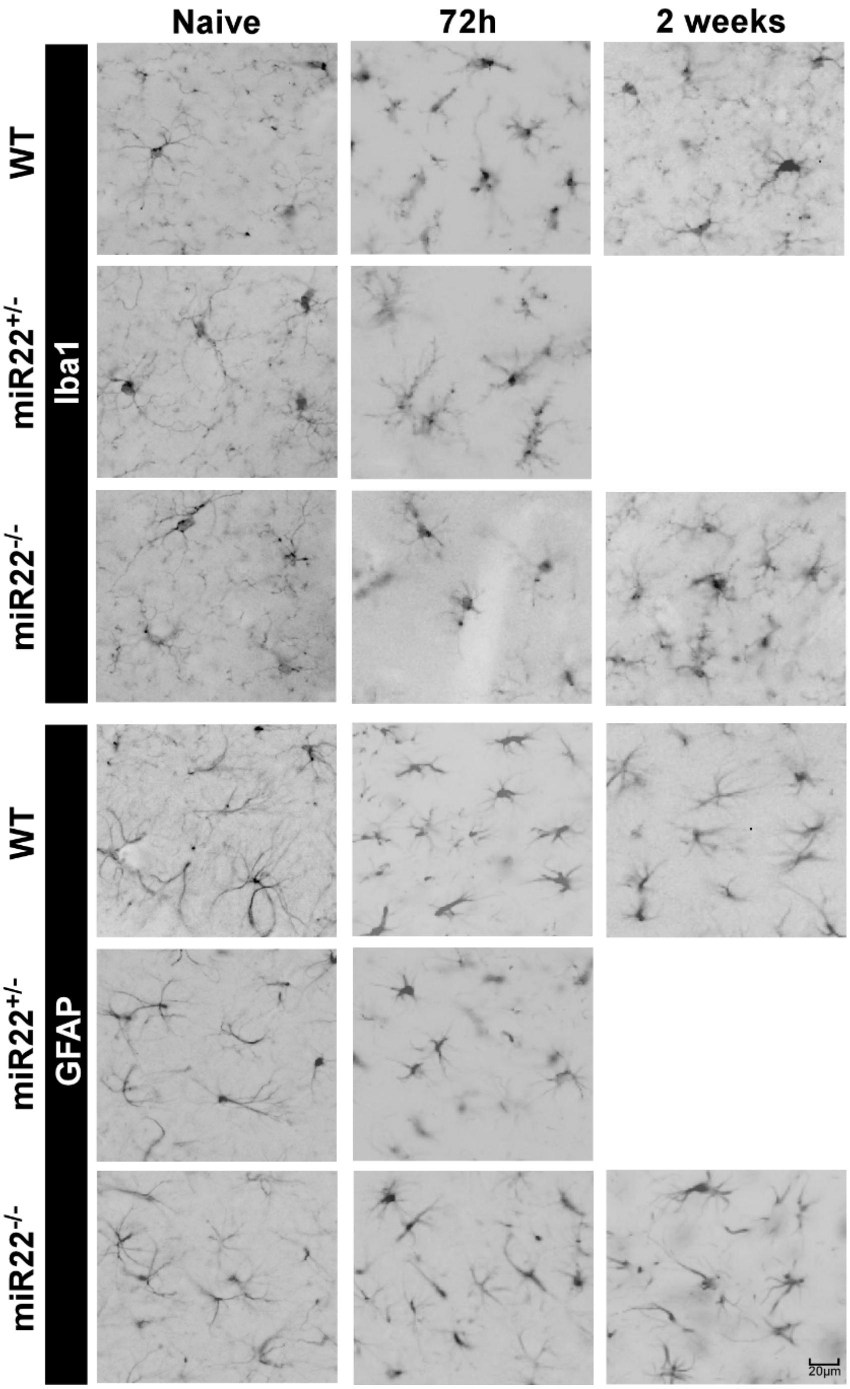

Supplementary Data Figure S6. *High-power images of glia in mice lacking miR-22*. Images show representative immunostaining from the ipsilateral CA3 subfield for microglia and astrocytes in the naïve brains, brains obtained 72 h after status epilepticus and the brains from mice at the end of monitoring.
